# Preliminary Outcomes of a Brief Values‐Guided Self‐Management Intervention to Improve Glucose Levels Among Emerging Adults With Type 1 Diabetes and Above‐Recommended HbA_1c_ : A Pilot Study

**DOI:** 10.1002/edm2.70255

**Published:** 2026-06-03

**Authors:** Sara E. Styles, Jillian J. Haszard, Zuzana Oravcova‐Wheeler, Anna Campbell, Bruce Arroll, Joseph Ciarrochi, Louise Hayes, Jim Lemon, Miriama Ketu‐McKenzie, Benjamin J. Wheeler

**Affiliations:** ^1^ Department of Human Nutrition University of Otago Dunedin New Zealand; ^2^ Haszard Biostatistics Balclutha New Zealand; ^3^ Zivio Health Dunedin New Zealand; ^4^ Te Whatu Ora – Health New Zealand (Southern) Dunedin New Zealand; ^5^ General Practice and Primary Healthcare, University of Auckland Auckland New Zealand; ^6^ Institute for Positive Psychology and Education, the Australian Catholic University North Sydney New South Wales Australia; ^7^ La Trobe University Melbourne Victoria Australia; ^8^ Department of Psychological Services and Research Mountainhall Treatment Centre Dumfries UK; ^9^ Ngāti Tūwharetoa and Ngāti Raukawa (Ki Horowhenua) Dunedin New Zealand; ^10^ Department of Psychology University of Otago Dunedin New Zealand; ^11^ Department of Paediatrics and Child Health University of Otago Dunedin New Zealand

**Keywords:** acceptance and commitment therapy, pilot study, self‐management, type 1 diabetes mellitus, young adult

## Abstract

**Introduction:**

A minority of young adults with type 1 diabetes achieve > 70% time in range (TIR; 70–180 mg/dL [3.9–10 mmol/L]), as recommended. Self‐management goals based on self‐chosen values (whatever is most important to an individual) may motivate engagement in self‐management tasks that could improve TIR. This study explored the acceptability, feasibility, and potential outcomes of values‐guided self‐management.

**Methods:**

A single‐arm quasi‐experimental pilot study was conducted with 14 participants aged 18–25 years and HbA_1c_ ≥ 58 mmol/mol (7.5%). The intervention consisted of a single 1:1 session (< 60 min) grounded in Acceptance and Commitment Therapy and a 2‐week follow‐up. The session consisted of a semi‐structured discussion of self‐management behaviour, behavioural alignment with personally chosen values, barriers to behaviour change, and action planning. TIR was determined by glucose levels measured with a blinded interstitial continuous glucose monitoring sensor. Participants self‐reported adherence to self‐management tasks, psychological flexibility, valued living and acceptance of living with diabetes.

**Results:**

Fourteen participants received the intervention. The intervention was acceptable and feasible to implement. The mean change (95% CI) between pre‐ and post‐intervention TIR was an increase of 2.2 (−1.2, 5.7) percentage points, mostly through a decrease in time above range. The standardized mean change (95% CI) in progress towards valued living was 0.72 (0.27, 1.16) with small improvements in diabetes‐specific acceptance and carrying out valued actions.

**Conclusions:**

A brief values‐guided self‐management intervention shows promise to improve time in range among emerging adults with type 1 diabetes and above recommended HbA_1c_. Further investigation of values‐guided self‐management is warranted.

**Trial Registration:**

Australian New Zealand Clinical Trials Registry (ACTRN12620000200987; https://www.anzctr.org.au/ACTRN12620000200987.aspx)

## Graphical abstract

After a single‐session values‐guided intervention, a significant increase in progress towards valued living was observed among emerging adults with type 1 diabetes and a higher than recommended HbA1c. However, this did not translate into an increase in the proportion of time that interstitial glucose levels were in the target range.

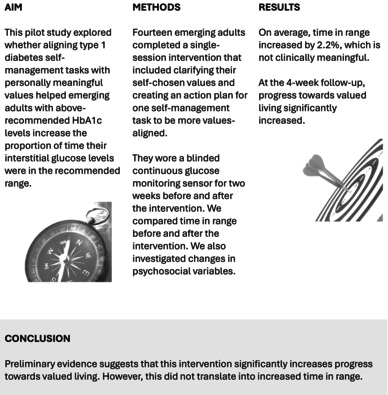

## 1 Introduction

Type 1 diabetes (T1D) is a chronic condition where maintaining glucose levels close to those in people without diabetes is key to reducing complications. The recommended glycated haemoglobin (HbA_1c_; average blood glucose) for nonpregnant adults is < 7.0% (< 53 mmol/mol), corresponding to 70% of interstitial glucose values from continuous glucose monitoring (CGM) between 70 and 180 mg/dL (3.9–10 mmol/L) [1]. Many emerging adults, aged 18 to < 25 years, and young adults, aged 25 to < 45 years, experience glucose levels above the recommended goals, with reasons including lower diabetes technology adoption, infrequent glucose level checks and missed insulin doses [2], transition to adult health care, moving away from home, shifts in supportive networks [3] and more. Effective interventions that support engagement in self‐management behaviours that underpin effective medical treatment are needed [4, 5].

Acceptance and Commitment Therapy (ACT) encourages a commitment to actions that align with a personally determined meaningful life guided by long‐term desired qualities, commonly known as values [6]. Integrating personal values into self‐management may motivate emerging adults to commit to and engage in self‐care as they navigate the challenges of adulthood [7, 8]. For example, someone who values social connection may experience a tension between monitoring glucose levels (which may support longer‐term wellbeing) and avoiding behaviours that disrupt social interactions. In some situations, they may choose to monitor and respond to glucose levels despite inconvenience, if they perceive this as subsequently supporting their ability to connect with others. This approach supports emerging adults' autonomy in engaging in self‐management tasks, which could improve short‐ and long‐term glycaemic outcomes and wellbeing [9]. ACT also focuses on developing psychological flexibility, a ‘critical skill’, underpinned by six behavioural regulation processes that encourage willingness to accept uncomfortable internal experiences when doing so supports values‐congruent behaviour [6, 8]. These processes are acceptance (i.e., allowing uncomfortable internal experiences to happen instead of controlling them), values, committed action, defusion (i.e., nonjudgmentally observing thoughts as just thoughts without getting ‘hooked’ by them), present moment awareness (i.e., mindfully noticing the here and now), and self‐as‐context (i.e., recognizing thoughts such as ‘I am a bad diabetic’ as thoughts rather than objective truths).

ACT‐based health‐related behaviour change interventions have been shown to be effective among emerging adults [10, 11, 12]. In a pilot randomized controlled trial, university‐aged participants identified a health domain to target (exercise, nutrition, or sleep). They were then randomly assigned to one of two conditions: (1) a single session ACT intervention that included clarifying values underpinning the desired change, co‐developing an action plan to link values and committed action explicitly, and strategies to overcome psychological and behavioural barriers to behaviour change; or (2) a comparison condition that provided information on how to improve health in the chosen domain. At 30 days post‐intervention, those in the ACT intervention group reported greater health‐related changes compared to those in the information‐only group, and improvements were maintained 60 days post‐intervention [11]. This approach of self‐selecting a domain to target for behaviour change support may be useful in a T1D context, which involves several self‐care domains, including glucose monitoring, medication, nutrition, exercise, sleep, and stress management. Cultivating awareness of the discrepancy between current self‐care and personally meaningful values or linking emerging adults' values to their behaviour change can elicit motivation for change [13], which might be a useful starting point for conversations in routine clinical care.

One clinical trial investigating four interventions among adolescents and emerging adults diagnosed with T1D (mean [SD] age 15.8 [2.3] years) found that a single‐session ACT‐based intervention [10] adapted for the T1D context, with a focus on values‐guided action planning, was more effective at increasing glucose levels in the clinically recommended range over 4 weeks than interventions that focused on CGM, healthier snacking, or sleep extension [14]. The study found time in range (TIR; i.e., the proportion of interstitial glucose values between 70 and 180 mg/dL [3.9–10 mmol/L]) increased by 6.1 (95% CI: −7.5, 19.7) percentage points from baseline to 4 weeks among participants who received the ACT intervention, a clinically meaningful change as defined by an increase of ≥ 5 percentage points [15], despite no significant increases in diabetes acceptance, valued living, or self‐care. Smaller improvements were observed in the CGM and snacking support intervention groups (3.3 [95% CI −8.8, 15.4] percentage points and 0.9 [95% CI −11.8, 13.5], respectively) and a deterioration was observed in the sleep group (−7.2 [−19.0, 4.6] percentage points). The results of the previous study suggest that a single session values‐guided self‐management intervention in adolescents and emerging adults could be effective for improving glycaemic outcomes. Furthermore, the previous study's values‐guided intervention protocol provides a simple, standardized approach that does not require extensive training to develop therapeutic competence of all ACT processes and is not time‐intensive to deliver.

This pilot study aimed to explore the preliminary effectiveness of a values‐guided self‐management intervention among emerging adults living with T1D. Findings will inform refinements before evaluation in a randomized controlled trial.

## 2 Methods

This quasi‐experimental pilot study used a single‐arm design. Consultation with Māori (Indigenous New Zealanders) was conducted to inform the study. Ethical approval was granted by the Southern Health and Disability Ethics Committee (20/STH/23; New Zealand). The trial was registered with the Australian New Zealand Clinical Trials Registry (ACTRN12620000200987; https://www.anzctr.org.au/ACTRN12620000200987.aspx) and assigned a Universal Trial Number (U1111‐1247‐4196) by the World Health Organization International Clinical Trials Registry.

### 2.1 Setting

The study was conducted in the Otago region of New Zealand, with recruitment from October 2020 to August 2022, which coincided with the COVID‐19 pandemic. Face‐to‐face visits were paused from 17 August to 6 September 2021 due to public health requirements, affecting one participant's intervention visit, two participants' assessment visits (contributing to missing self‐reported data), and three participants' final visits.

### 2.2 Participants and Recruitment

A target sample size of 20 participants was deemed appropriate to estimate the mean and standard deviation (SD) for the change in TIR (i.e., glucose levels in the clinically recommended range of 70–180 mg/dL [3.9–10 mmol/L]) to inform future trial design. Inclusion criteria were age 18–25 years, HbA1c ≥ 7.5% (≥ 58 mmol/mol) within 14 days of enrolment, and English proficiency. Exclusion criteria included severe, uncontrolled comorbidities or participation in studies that affected glucose levels. Recruitment occurred during routine diabetes clinic visits. Healthcare providers identified potential participants, provided a study overview, and shared a Participant Information Sheet.

### 2.3 Procedures

Enrolment followed written informed consent and confirmation of diagnosis, age, and recent HbA_1c_. HbA_1c_ was measured by a calibrated DCA Vantage Analyser (Siemens Healthcare Diagnostics, Dublin, Ireland), linked directly to the Diabetes Control and Complications Trial method (certified through the National Glycohemoglobin Standardization Program) [16].

The study included four visits, spaced 14 days apart, conducted via Zoom or in a hospital clinic. The intervention was delivered at the second study visit. Glycemic metrics were collected for up to 14 days pre‐ and post‐intervention. Secondary outcomes were assessed using validated self‐report questionnaires at pre‐intervention and at 2‐ and 4‐weeks post‐intervention. Data were managed on REDCap, a secure electronic platform hosted at the University of Otago [17]. Participants received a NZ$20 (US$12) gift voucher after their final study visit.

### 2.4 Values‐Guided Self‐Management Intervention

Experts in diabetes (BW), Māori health (MKM), ACT in general practice (BA, ZOW, JL), adolescent and adult mental health (AC, JC, LH) and advisory board members (emerging adults with diabetes and prior research participation experience) provided input into the intervention protocol, which was subsequently feasibility tested before commencing this pilot study. In the feasibility study, five emerging adults living with T1D received the intervention and rated its helpfulness on a scale from 1 (extremely unhelpful) to 5 (extremely helpful), with 4 participants rating it 4/5 and 1 participant rating it 5/5. During feasibility testing, a researcher trained in ACT assessed fidelity by observing sessions and comparing delivered content with the protocol, without any issues identified; therefore, fidelity was not assessed in the present study.

The single‐session 1:1 intervention involved a semi‐structured discussion lasting 30 to 60 min, delivered by a non‐medical interventionist (i.e., a health coach who completed introductory and intermediate ACT workshops). Participants clarified values through an electronic card‐sorting task. They sorted cards labelled with values into three categories (very important, important, less important right now). One deck featured Western values with English labels and definitions [18], while another comprised Māori (Indigenous New Zealander) values with te reo Māori (the language of Indigenous New Zealanders) labels and English definitions [19]. Action planning was completed using a modified health‐related ACT matrix [10, 20] (see Appendix S1), focusing on diabetes self‐management. Participants recorded self‐management behaviours they wanted to change, how the behaviours aligned with one of their values, potential internal obstacles and behavioural barriers, and detailed action plans for 14 days, including tasks to complete within 24 h to support behaviour change. A handout informed by the Discoverer, Noticer, Advisor‐Values model [21] provided ACT‐based coping strategies for psychological barriers. A kaumātua (Māori elder) translated key messages into te reo Māori (see Appendix S1). Participants identified at least one coping strategy to support the completion of their action plan. At a 2‐week follow‐up, participants reviewed their goals. Those achieving the behaviour change continued their plans, while others received support to modify their action plan for the final 2 weeks.

### 2.5 Measures

#### 2.5.1 Participant Characteristics

Gender, ethnicity, insulin regimen, and address were self‐reported. Participants' addresses determined New Zealand Deprivation Index 2018 (NZDep18) scores, a socioeconomic deprivation measure with scores split into deciles numbered from 1 (lowest deprivation) to 10 (highest deprivation) [22]. The Patient Health Questionnaire (PHQ‐9) [23] screened for depression. Participants scoring 15+ (moderately severe to severe depression) were offered mental health referrals. The Diabetes Distress Scale (DDS2) [24] measured pre‐intervention distress.

#### 2.5.2 Glycemic Metrics

The primary outcome was the mean change in TIR. Participants wore a blinded CGM sensor (FreeStyle Libre Pro, Abbott) for 14 days before the intervention and between the 2‐ and 4‐week follow‐ups. Time above and below the recommended range and mean interstitial glucose levels were also recorded. Four participants using CGM at enrollment continued using their usual CGM system and wore the additional blinded CGM sensors provided by the study.

#### 2.5.3 Psychosocial Outcomes

Validated instruments were administered to assess diabetes self‐management and measure psychological flexibility as a potential mechanism of change.

The 15‐item Self‐Care Inventory‐Revised (SCI‐R) [25] measured adherence to diabetes self‐management. Participants self‐reported adherence to diabetes self‐care recommendations over the past month. Responses, rated on a 5‐point Likert scale from 1 (‘never’) to 5 (‘always’), yield scores ranging from 0 to 100. Higher scores indicate higher levels of self‐care.

The 10‐item Valuing Questionnaire (VQ) [26] measured valued living. Two subscales measure progress in valued living (e.g., I worked towards my goals even if I didn't feel motivated to) and obstruction to valued living (e.g., When things didn't go according to plan, I gave up easily). Participants self‐reported how much a statement was true during the past week on a 7‐point Likert scale ranging from 0 (‘not at all true’) to 6 (‘completely true’). Subscale scores range from 0 to 30, with higher progress scores indicating closer alignment between one's personally chosen values and actions, and higher obstruction scores indicating barriers to valued living and obstruction to valued living.

The 9‐item Diabetes Acceptance and Action Scale‐Revised (DAAS‐R) [27] measured diabetes‐specific psychological flexibility. Participants self‐reported disruption of valued living due to diabetes (e.g., diabetes stops me from doing what I want to) on a 5‐point Likert scale from 0 (‘never true’) to 4 (‘not at all true’). All items were reverse‐scored and summed to calculate a total DAAS‐R score. Scores range from 0 to 36. Higher scores suggest greater acceptance of diabetes and the ability to perform values‐consistent actions despite challenges related to diabetes.

The 23‐item Comprehensive Assessment of Acceptance and Commitment Therapy processes (CompACT) [28] measured psychological flexibility, with three subscales measuring (1) openness to experience (e.g., ‘My thoughts are just thoughts – they don't control what I do’), (2) behavioural awareness (e.g., ‘I do jobs or tasks automatically, without being aware of what I'm doing’), and (3) valued action (e.g., ‘I can identify the things that really matter to me in life and pursue them’). Responses were rated on a 7‐point Likert scale from 0 (‘strongly disagree’) to 6 (‘strongly agree’). A CompACT Total score was calculated as the sum of subscale scores and ranges from 0 to 138, with higher scores indicating a tendency to be more psychologically flexible.

#### 2.5.4 Adverse Events

Participants self‐reported adverse events, including cutaneous issues from CGM sensors, severe hypoglycemia, diabetic ketoacidosis or mental health‐related events. Given the behavioural nature of the intervention and absence of changes to clinical management, no adverse events were considered likely to be attributable to the study procedures.

#### 2.5.5 Intervention Evaluation

Participants completed an electronic evaluation at the final visit, sharing what they liked most and least about the intervention. Data was coded inductively to identify categories [29], which are summarized in Appendix S1 and presented with related participant quotes.

### 2.6 Statistical Analysis

Mean changes between pre‐ and post‐ intervention were determined for all primary and secondary measures with standard deviations and 95% confidence intervals. Missing data were excluded list‐wise. All analyses were undertaken using Stata 18.0 (StataCorp, Texas) by a biostatistician (JH).

## 3 Results

### 3.1 Participant Recruitment and Retention

**FIGURE 1:**
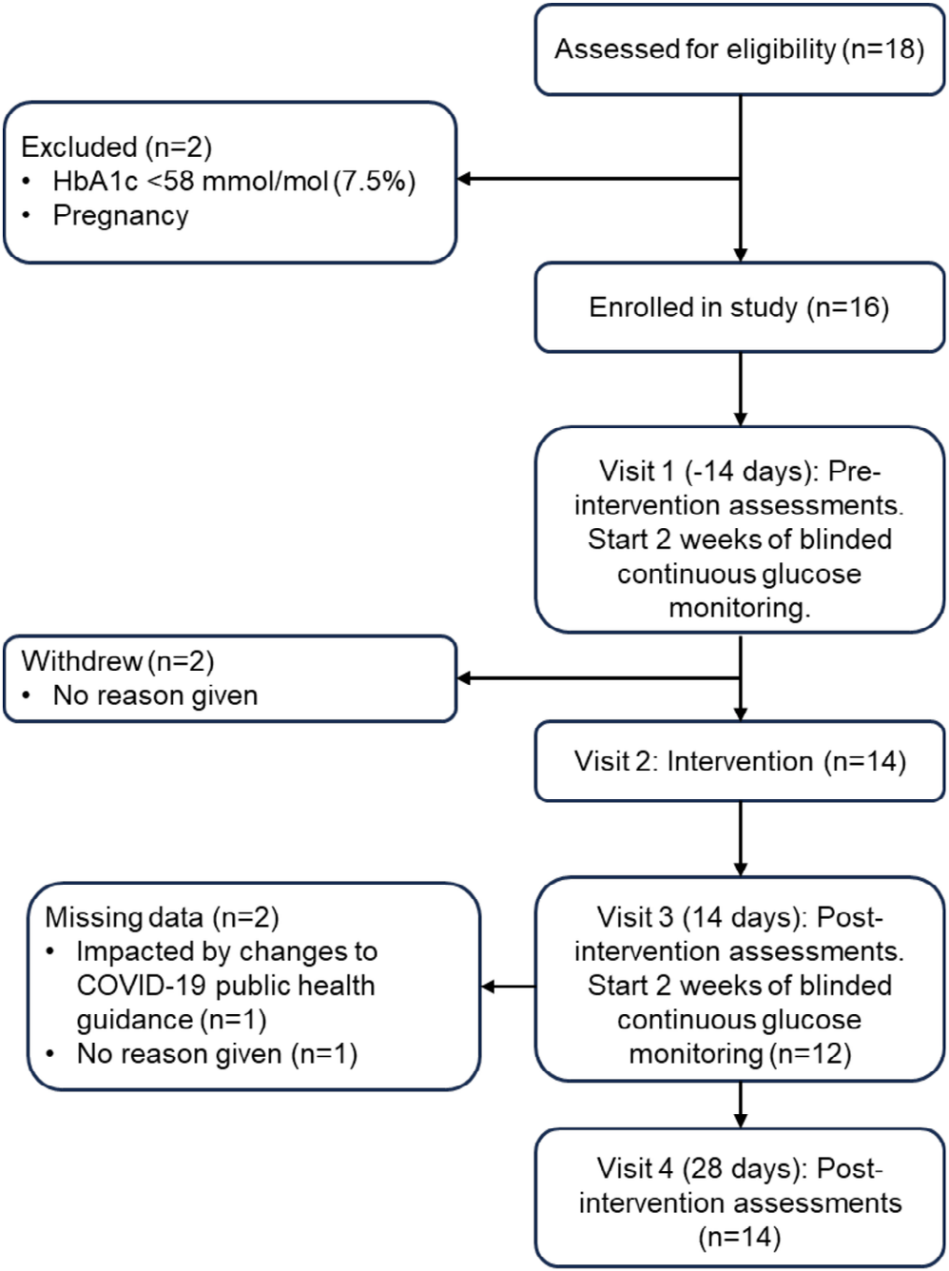
Participant flow from recruitment to the 4‐week follow‐up.

Figure 1 presents the participant flow from recruitment through the 4‐week follow‐up. A recruitment rate was not calculated due to missing data on the number of potential participants approached, as diabetes care providers were working under challenging and evolving conditions during the COVID‐19 pandemic. In total, 18 potential participants were assessed for eligibility. One participant was ineligible due to an HbA1c below the minimum criteria, and another was ineligible due to pregnancy. Sixteen participants were enrolled. Table 1 shows the baseline characteristics of the sample. Of these, 14 participants received the intervention, and 2 participants withdrew (no reason given) before receiving the intervention. Twelve participants completed all assessments.

**TABLE 1:** Participant baseline characteristics (*n* = 14).

	Mean (SD) or *n* (%) as specified
Age, mean (SD) years	21.1 (1.9)
Gender, *n* (%)	
Female	12 (85.7)
Male	2 (14.3)
Ethnicity^a^, *n* (%)	
New Zealand European	10 (71.4)
Other	4 (28.6)
Deprivation level^b^, *n* (%)	
1–3 (Low)	6 (42.9)
4–7	2 (14.3)
8–10 (High)	6 (42.9)
Duration since T1D diagnosis^c^, mean (SD) years	10.2 (4.5)
HbA_1c_ ^d^, mean (SD) %	9.1 (1.3)
HbA_1c_ ^d^, mean (SD) mmol/mol	76 (14.2)
Insulin regimen, *n* (%)	
Insulin pump	8 (57.1)
MDI	6 (42.9)
Continuous glucose monitoring use at enrolment	
Yes, *n* (%)	4 (28.6)
Depression (PHQ‐9; screening)^e^, mean (SD)	8.5 (7.0)
Diabetes Distress (DDS2; screening)^f^, Mean (SD)	3.6 (1.1)

^a^ Ethnicity prioritized as ‘Other’ if specified. ‘Other’ ethnicities were: Cook Island Māori, Indian, African and European.

^b^ Household deprivation assessed by home address applied to the New Zealand Deprivation Index 2018, with higher deciles indicating higher deprivation.

^c^ Duration since T1D diagnosis ranged from 1.8 to 16.1 years.

^d^ HbA1c ranged from 7.0% to 11.9% (53–107 mmol/mol).

^e^ Depression was measured on a 9‐item scale from 0 to 27, with higher scores indicating more depressive symptoms. Scores ranged from 0 to 21.

^f^ Diabetes Distress Screening Scale measured on a 2‐item scale from 1 to 6, with higher scores indicating more distress. Scores ranged from 2 to 5.5.

### 3.2 Clinical Outcomes

Table 2 presents the glycemic outcomes of the study. TIR increased by 2.2 (95% CI: −1.2, 5.7; SD: 6.0) percentage points from 33.8% (SD: 18.3) pre‐intervention to 36.0% (SD: 17.1) post‐intervention. Over half of the participants (57%, 8/14) experienced a clinically relevant change in TIR (an increase of > 5 percentage points [15]). Time above range decreased, on average, by 2.6 (95% CI: −7.8, 2.6) percentage points and time below range increased by 0.4 (95% CI: −2.5, 3.3) percentage points. Mean interstitial glucose was modestly lower post‐intervention (mean change [95% CI] between pre‐ and post‐intervention −0.3 mmol/L [−1.3, 0.6] (−5.5 mg/dL [95% CI: −23.4, 10.8])). Public health guidelines in response to the COVID‐19 pandemic prevented staff from downloading all participants' glucose monitoring data. Therefore, capillary blood glucose monitoring frequency is not reported due to incomplete data for most participants. There were no adverse events reported.

**TABLE 2:** Glycemic outcomes for the pre–post intervention change (*n* = 14).

	Mean (SD) pre‐intervention	Mean (SD) post‐intervention	Standardized^a^ mean change (95% CI) between pre‐ and post‐intervention
Percentage of time with glucose in target (70–180 mg/dL [3.9–10.0 mmol/L]) range	33.8 (18.3)	36.0 (17.1)	2.2 (−1.2, 5.7)
Percentage of time with glucose above 180 mg/dL (10.0 mmol/L)	63.2 (19.2)	60.6 (18.9)	−2.6 (−7.8, 2.6)
Percentage of time with glucose below 70 mg/dL (3.9 mmol/L)	3.0 (4.7)	3.4 (2.9)	0.4 (−2.5, 3.3)
Interstitial glucose level, mg/dL	232.4 (68.0)	227.0 (74.0)	−5.4 (−23.4, 10.8)
Interstitial glucose level, mmol/L	12.9 (3.8)	12.6 (4.1)	−0.3 (−1.3, 0.6)

^a^ Standardized mean change determined using a pooled standard deviation.

### 3.3 Psychosocial Outcomes

Table 3 presents the study's psychosocial findings. Unstandardized mean changes are reported in Table S1. Mean changes were relatively small across all psychosocial outcomes, except for the progress subscale of the valuing questionnaire. Behavioural progress towards the enactment of personally important values changed by a standardized mean difference of 0.72 (95% CI: 0.27, 1.16) with the mean Valuing Questionnaire progress subscale score increasing from 16.0 (SD = 6.6) at baseline to 20.2 (SD = 5.7) at 4 weeks post‐intervention. Obstruction of valued living changed by a standardized mean difference of −0.22 (95% CI: −0.76, 0.32) with the mean Valuing Questionnaire obstruction subscale score decreasing from 14.1 (SD = 6.7) at baseline to 12.5 (SD = 7.4) at 4 weeks post‐intervention. Diabetes‐specific psychological flexibility changed by a standardized mean difference of 0.38 (95% CI: −0.22, 0.98), with the mean DAAS‐R score increasing from 41.5 (5.5) at baseline to 43.4 (SD = 5.4) at 4 weeks post‐intervention. General psychological flexibility increased by a standardized mean difference of 0.18 (95% CI: −0.16, 0.53), with the mean CompACT total score increasing from 64.1 (SD = 25.3) at baseline to 69.1 (SD = 29.4) at 4 weeks post‐intervention. Values and committed action increased by a standardized mean difference of 0.26 (95% CI: −0.14, 0.66) with the mean CompACT valued action subscale score increasing from 33.5 (SD = 8.6) at baseline to 35.6 (SD = 8.4) at 4 weeks post‐intervention. Mean changes across the CompACT subscale scores followed a similar trend of improving from baseline to 4 weeks post‐intervention. Adherence to self‐management changed by a standardized mean difference of 0.18 (95% CI: −0.15, 0.51) with the mean SCI‐R score increasing from 56.0 (SD = 13.0) at baseline to 58.0 (SD = 11.0) at 4 weeks post‐intervention.

**TABLE 3:** Psychosocial outcomes for the pre–post intervention change (*n* = 14).

	Mean (SD) at baseline	Mean (SD) at 2‐wk (post‐ intervention)	Mean (SD) at 4‐wk (post‐intervention)	Standardized^a^ mean change (95% CI) between baseline and 2 weeks	Standardized^a^ mean change (95% CI) between baseline and 4 weeks
*n*	14	12	14	12	14
Self‐management (SCI‐R)^b^	56 (13.0)	56 (12.0)	58 (11.0)	−0.01 (−0.30, 0.28)	0.18 (−0.15, 0.51)
Valued living^c^: Progress	16.0 (6.6)	16.8 (4.5)	20.2 (5.7)	0.04 (−0.38, 0.46)	**0.72 (0.27, 1.16)**
Valued living^c^: Obstruction	14.1 (6.7)	13.5 (8.0)	12.5 (7.4)	−0.01 (−0.34, 0.32)	−0.22 (−0.76, 0.32)
Diabetes‐specific acceptance^d^ (DAAS‐R)	41.5 (5.5)	42.0 (4.0)	43.4 (5.4)	0.12 (−0.54, 0.77)	0.38 (−0.22, 0.98)
Psychological flexibility (CompACT)^e^: openness to experience	14.2 (12.4)	16.3 (15.8)	16.9 (15.2)	0.09 (−0.21, 0.39)	0.19 (−0.15, 0.53)
Psychological flexibility (CompACT)^e^: behavioural awareness	16.4 (7.8)	16.4 (9.0)	16.6 (8.6)	−0.05 (−0.28, 0.18)	0.02 (−0.36, 0.39)
Psychological flexibility (CompACT)^e^: valued action	33.5 (8.6)	34.1 (8.3)	35.6 (8.4)	0.16 (−0.04, 0.36)	0.26 (−0.14, 0.66)
Psychological flexibility (CompACT)^e^: total score	64.1 (25.3)	66.8 (29.6)	69.1 (29.4)	0.08 (−0.13, 0.29)	0.18 (−0.16, 0.53)

^a^ Standardized mean changes determined using a pooled standard deviation. Bold font indicates that the 95% confidence interval for the standardized mean change does not include 0. Unstandardized mean changes are reported in Table S2.

^b^ Self‐management scored on a scale between 0 and 100, with higher scores indicating higher levels of self‐care.

^c^ Valued living subscales scored on a scale from 0 to 30, with higher scores indicating either greater progress at living within one's values (Progress) or greater disruptions to living one's values (Obstruction).

^d^ Diabetes acceptance scored on a scale between 0 and 54, with higher scores indicating greater acceptance and ability to take action with diabetes‐related challenges.

^e^ The CompACT scale has three subscales: openness to experience scored on a scale from 0 to 60; behavioural awareness scored on a scale from 0 to 30; and valued action scored on a scale from 0 to 48. The total score is on a scale from 0 to 138. Higher scores indicate greater psychological flexibility.

### 3.4 Intervention Evaluation

Participant feedback is presented in Table S2. In summary, participants evaluated the intervention favourably because it was tailored to the individual (i.e., the intervention focused on what was important to the individuals and not focused on ‘the numbers’, was based on current experiences and thoughts about diabetes‐specific situations, the behaviour change goal was achievable), which was perceived to be more likely to contribute to long‐term behaviour change because changes were ‘within one's control’. Additionally, the interventionist was nonjudgmental towards glucose levels, the incentive for managing diabetes was framed more positively (i.e., in contrast to ‘scare tactics’ about ‘bad’ glucose levels), participants' autonomy and self‐efficacy were supported, and strategies for coping with difficult thoughts were useful. Participants reported that clarifying values felt ‘intense, but important’ (i.e., reflecting on the gap between current self‐management behaviours and ‘ideal’ self‐management contributed to feeling disappointment towards oneself or overwhelmed), identifying feelings in order to verbalize them was difficult, and remembering to follow the action plan was difficult at first, but ‘became a routine with time’.

## 4 Discussion

This pilot study aimed to quantify the impact of a values‐guided self‐management intervention on time in range (TIR) among emerging adults with an HbA1c ≥ 7.5% (≥ 58 mmol/mol). Following the single‐session intervention, over half of the participants experienced a clinically meaningful improvement in TIR; however, the improvement in TIR and other glycemic metrics (i.e., time above range, mean interstitial glucose level) were not significant. Similarly, there were small improvements in psychosocial outcomes (self‐management, valued living, psychological flexibility), however, the only statistically significant improvement was progress towards valued living. In general, participants viewed the intervention favourably.

Previous research has shown that a brief single‐session values‐guided self‐management intervention was associated with a clinically meaningful increase in TIR among adolescents and emerging adults [14]; however, in the present study, TIR increased by 2.2 percentage points, which was not a clinically meaningful change [15]. The 95% confidence interval for the change in TIR (−1.2%, 5.7%) includes values consistent with both a decrease and a clinically meaningful increase in TIR, suggesting equivocal results. This may be related to the present study's sample characteristics, including older age contributing to navigating more complexities (e.g., work, university, independence), lower baseline TIR (potentially due to more self‐management independence, differences in lifestyle factors, lower uptake of CGM), and higher self‐reported baseline depression [3]. Heterogeneity in outcomes, eligibility criteria and study designs makes comparisons across other study outcomes difficult across the few other ACT‐based diabetes intervention trials [30, 31, 32, 33, 34]. Ultimately, a larger sample size and a control group are needed to investigate the potential for a clinically meaningful effect on TIR following a single‐session values‐guided self‐management intervention among emerging adults with above recommended HbA1c.

There was evidence of a significant increase in the progress subscale of the valuing questionnaire, suggesting participants made progress in valued life domains between baseline and 4 weeks post‐intervention. Yet, it's uncertain whether this progress in valued life domains was specific to diabetes‐specific changes that were not assessed in the questionnaires (i.e., making small changes to self‐care) or other personally meaningful domains (e.g., school, work, relationships, sports). Although this improvement did not translate to significant changes in self‐management or glycaemic metrics, it may reflect early changes in valued living that had not yet translated into measurable clinical or diabetes‐specific behavioural outcomes. Based on previous research, it is unclear whether a longer intervention that includes all ACT processes could translate into improvements in glycaemic metrics in our clinical population of interest, given differences in sample characteristics and interventions. Somaini, Kingston & Taylor [31] investigated the preliminary effectiveness of a 6‐module online ACT intervention for adults (mean age 40.9 years [SD = 9.48]). TIR was available for 4 of 9 participants, all of whom experienced an increase in TIR during the 6‐week intervention phase, but this increase was not sustained at the 4‐week follow‐up. Other studies investigating the effectiveness of ACT‐based interventions have not reported CGM metrics. In a randomized controlled trial among adults aged 18–70 years with an HbA1c > 7.6% (> 60 mmol/mol), Wijk et al. [32] found no significant difference in HbA1c between the ACT intervention group, which received seven 2‐h group sessions incorporating all ACT processes, and the treatment‐as‐usual control group at any time point over 2 years. Conversely, in a pilot study of adolescents aged 12–16 years with an HbA1c > 7.5% (> 58 mmol/mol), Alho et al. [30] reported that, at 3 months, the ACT intervention group, which received 5 1.5‐h group sessions informed by all ACT processes, showed a significantly greater improvement in HbA1c compared with the treatment‐as‐usual control group. A qualitative or mixed‐methods design exploring barriers and facilitators to values‐guided self‐management change following a single‐session values‐guided intervention, which could inform future improvements.

Small improvements across obstruction to valued living, diabetes‐specific acceptance, and psychological flexibility were observed. This provides preliminary evidence that the intervention helps emerging adults cope with psychological challenges and aligns with other underlying ACT processes. The non‐significant improvement in self‐reported self‐management may be explained by emerging adults feeling more motivated to change their self‐management behaviours, but additional sessions to review goals, revise action plans and address barriers such as financial constraints, racial/ethnic influences [35], self‐ and social‐regulation [36] might be needed to support further engagement in self‐management tasks. Indeed, qualitative findings from this study provide evidence that the intervention is an acceptable and personalized approach to clinical care. These results indicate that clarifying personally meaningful values and identifying ways to enact them in diabetes self‐management could be an important strategy for fostering motivation among emerging adults with HbA1c levels above recommended targets. Given that the intervention was simple and would require minimal training to implement collaborative action planning, it has potential utility on a wider scale.

Strengths of this study include a theoretically informed intervention developed by a multidisciplinary team, that was reviewed by emerging adults living with type 1 diabetes who had prior research participation experience and feasibility tested prior to the pilot study, and the use of blinded CGM to measure the primary outcome. Limitations include the single‐arm design, short duration, and pilot study objectives (i.e., the study is underpowered for hypothesis testing). Recruitment and retention were affected by the COVID‐19 pandemic, particularly among Māori [37]. Pandemic‐related stressors (e.g., social isolation, financial burden, COVID‐19 infection in self or close relations) may underestimate the intervention's effects and limit the generalizability of the findings. Recruitment bias may have favoured participants with fewer barriers to participation or an interest in psychoeducational support. The majority of participants were female, which is likely due to females self‐reporting higher levels of diabetes distress [38]. Further research is needed to investigate whether the findings are generalizable to males. Lastly, self‐reported measures may have introduced social desirability bias. A randomized controlled trial with a larger sample size, an evidence‐based active treatment comparison group, and longer follow‐up would provide more robust evidence of the effectiveness of this intervention.

## 5 Conclusion

This pilot study provides preliminary evidence that this single‐session values‐guided self‐management intervention improves progress towards valued living among emerging adults with T1D and above recommended HbA1c.

## Author Contributions

**Sara E. Styles:** conceptualization, data curation, funding acquisition, investigation, methodology, project administration, resources, writing – original draft, writing – review and editing. **Benjamin J. Wheeler:** methodology, funding acquisition, resources, writing – review and editing. **Louise Hayes:** resources, writing – review and editing. **Jillian J. Haszard:** methodology, formal analysis, funding acquisition, writing – review and editing. **Anna Campbell:** resources, writing – review and editing. **Zuzana Oravcova‐Wheeler:** methodology, investigation, writing – review and editing. **Miriama Ketu‐McKenzie:** resources, writing – review and editing. **Bruce Arroll:** resources, writing – review and editing. **Jim Lemon:** writing – review and editing, resources. **Joseph Ciarrochi:** resources, writing – review and editing.

## Funding

Healthcare Otago Charitable Trust provided financial support for the study. The funder was not involved in the study planning, design, implementation, or reporting.

## Ethics Statement

Approval was granted by the Southern Health and Disability Ethics Committee (20/STH/23).

## Conflicts of Interest

The authors declare no conflicts of interest.

## Supporting information


**Table S1:** Unstandardized psychosocial changes.
**Table S2:** Participants' feedback about the values‐guided self‐management intervention.

## Data Availability

The data that support the findings of this study are available on request from the corresponding author. The data are not publicly available due to privacy or ethical restrictions.
