# Supplementary material for: Preliminary Outcomes of a Brief Values‐Guided Self‐Management Intervention to Improve Glucose Levels Among Emerging Adults With Type 1 Diabetes and Above‐Recommended HbA1c : A Pilot Study
Source: Endocrinol Diabetes Metab. 2026 Jun 3;9(4):e70255. doi: 10.1002/edm2.70255 (PMC13239978; doi:10.1002/edm2.70255)
Supplement: Supplementary file 1 — Table S1: Unstandardized psychosocial changes. Table S2: Participants' feedback about the values‐guided self‐management intervention. [file EDM2-9-e70255-s001.docx]

**Appendix S1.** Intervention handout #1

All of what you’ve been told to take care of your diabetes will only work if you do the things that you need to do to be healthy.

Although you don’t have a choice about having diabetes, you get to choose who you will take care of yourself. The activity below will guide you through choosing a diabetes-related behaviour to work on and how you will put change into action.

Importantly, you’ll identify what you think and do that might move you away from making a change. Check out the Strategies for Overcoming Common Motivational and Psychological Barriers handout for ideas to deal with your unhelpful thoughts and behaviours.

STEP 1: Answer this question: What problem with my diabetes would I like help with?

STEP 2: Starting with the Values box and, moving clock-wise, answer all questions below.


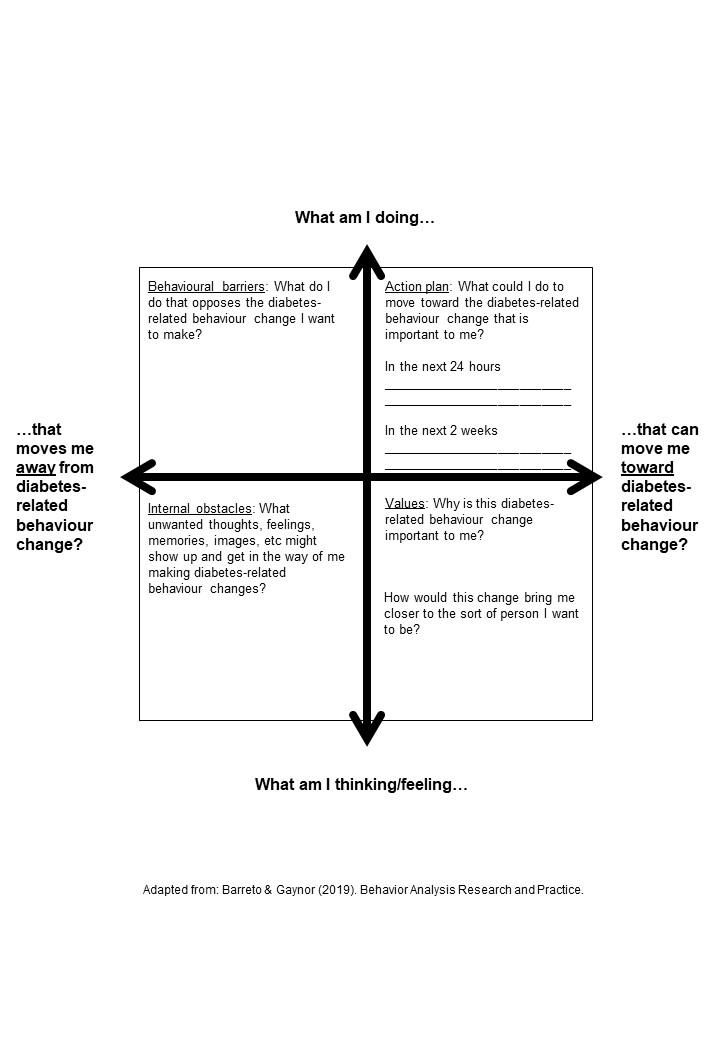


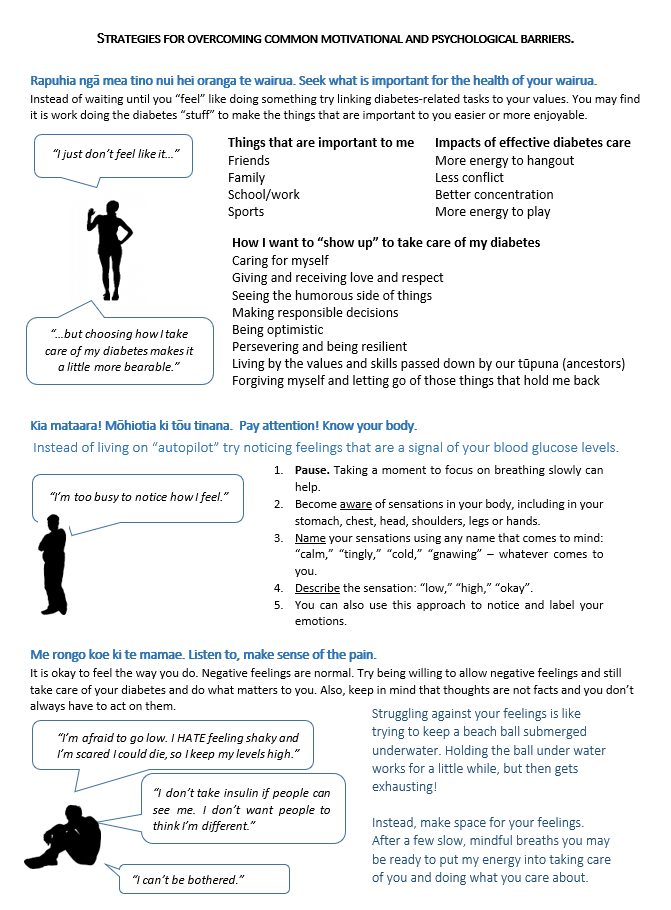


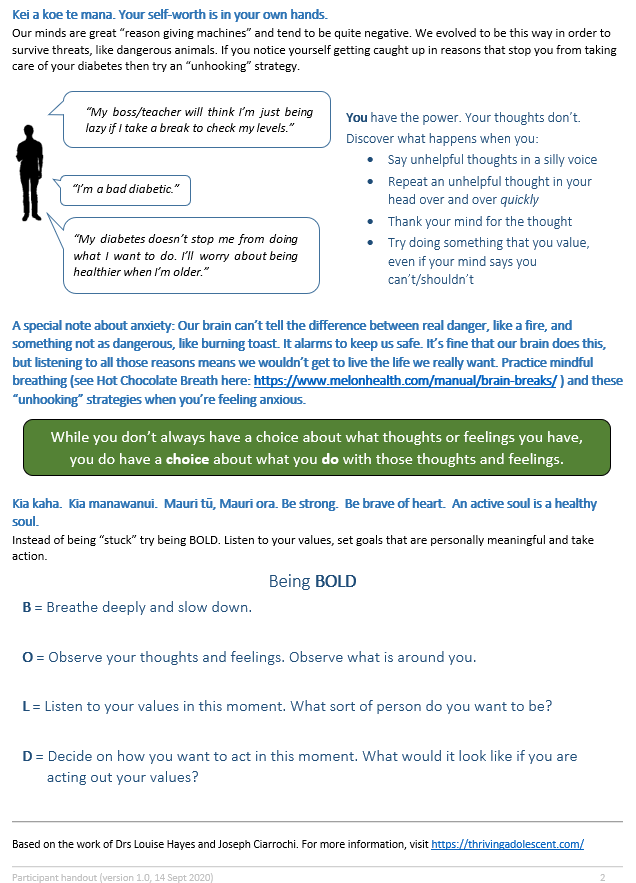


**Supplementary Table 1.** Unstandardized psychosocial changes

|  | Mean change between baseline and 2 weeks (95% CI) | Mean change between baseline and 4 weeks (95% CI) |
| --- | --- | --- |
| n | 12 | 14 |
| Self-management (SCI-R score)^a^ | -0.1 (-3.6, 3.3) | 2.1 (-1.8, 6.1) |
| Valued living^b^: Progress | 0.3 (-2.2, 2.7) | **4.2 (1.6, 6.8)** |
| Valued living^b^: Obstruction | -0.1 (-2.4, 2.3) | -1.6 (-5.4, 2.3) |
| Diabetes-specific acceptance^c^ (DAAS-R) | 0.6 (-2.7, 3.9) | 1.9 (-1.1, 4.9) |
| Psychological flexibility (CompACT)^d^: openness to experience | 1.3 (-3.0, 5.5) | 2.7 (-2.1, 7.5) |
| Psychological flexibility (CompACT)^d^: behavioral awareness | -0.4 (-2.3, 1.5) | 0.1 (-3.0, 3.2) |
| Psychological flexibility (CompACT)^d^: valued action | 1.3 (-0.3, 3.0) | 2.1 (-1.2, 5.4) |
| Psychological flexibility (CompACT)^d^: total score | 2.2 (-3.5, 7.9) | 4.4 (-4.5, 14.5) |

^a^ Self-management scored on a scale between 0 and 100, with higher scores indicating higher levels of self-care

^b^ Valued living subscales scored on a scale from 0 to 30, with higher scores indicating either greater progress at living within one’s values (Progress) or greater disruptions to living one’s values (Obstruction).

^c^ Diabetes acceptance scored on a scale between 0 and 54, with higher scores indicating greater acceptance and ability to take action with diabetes related challenges.

^d^ The CompACT scale has three subscales: openness to experience, scored on a scale from 0 to 60; behavioral awareness, scored on a scale from 0 to 30; and valued action, scored on a scale from 0 to 48. The total score ranges from 0 to 138. Higher scores indicate greater psychological flexibility.

**Supplementary Table 2.** Participants' feedback about the values-guided self-management intervention

| Research questions | Key feedback |
| --- | --- |
| What did you like MOST about the intervention? | **Tailored to the individual**  “The goal wasn’t over-ambitious. It was achievable.”  “The intervention focused on something besides the numbers (glucose levels).”  “I liked the format of generating an intervention based on my current experiences and thoughts about the situations.”  “The incentive for managing diabetes was more positive instead of using scare tactics about “bad” glucose levels and the risk for severe long-term diabetes complications”  **Nonjudgemental approach**  “There was no judgment about numbers (glucose levels).”  **Useful**  “Strategies (were provided) to cope with negative thoughts.”  “I would totally love to do this type of study again or at least see it being used in clinics”  **Autonomy supporting**  “(the intervention) made me think about how I could be better if I managed my diabetes better and ‘better’ was up to me.”  **Holistic approach**  “(the intervention) focused on what was actually important and had the potential to make a long-term change.”  **Self-efficacy enhancing**  “Verbalizing what was already known about self-management contributed to motivation to make changes within one’s control.” |
| What did you like LEAST about the intervention? | **Talking about emotions around diabetes is difficult.**  “I am terrible at expressing my feelings”  **Clarifying values might be “intense” but important.**  “… warn participants about this intensity in advance so they have time to think about their values before the intervention visit”  **Remembering to follow the action plan was difficult at first, but became a routine with time**  “Remembering to do the intervention initially was difficult, but that's also a testament to my absolutely shocking memory. But once I got into the routine of checking in with myself, it got much more normal.” |
| Other | **COVID-19 pandemic disruption**  “I think it was going really well until we went into lock-down again, and I noticed I was stress eating a little more, and exercising a lot less, which may have affected the last weeks worth of data. But I am definitely going to keep on in the future and check in with how I'm feeling and whether I need to act on it in the future” |
|  | **Self-identifying strategies to remember values**  “… following the values chat I designed a phone case that kinda represents the value I found important in an attempt to remind me to test more often so we'll see if that works” |
